# Beyond Distraction: Clinical Case of ADHD in a 12-Year-Old Boy

**DOI:** 10.1192/j.eurpsy.2026.11129

**Published:** 2026-07-31

**Authors:** I. M. Peso Navarro, C. Garcia Cerdan, M. Ligero Argudo, M. Bersabe Perez, M. Covacho Gonzalez, C. Munaiz Cossio

**Affiliations:** 1 Psiquiatria, Complejo Asistencial Universitario de Salamanca, Salamanca; 2 Psicología, Hospital Gregorio Marañon, Madrid, Spain

## Abstract

**Introduction:**

Attention-Deficit/Hyperactivity Disorder (ADHD) is one of the most common neurodevelopmental disorders in childhood and adolescence. It is characterized by pervasive patterns of inattention, hyperactivity, and impulsivity that significantly interfere with academic, social, and family functioning. Early identification and individualized treatment are key to improving long-term outcomes.

**Objectives:**

To present the clinical case of a 12-year-old boy with ADHD.

To describe his developmental, academic, and social functioning.

To discuss therapeutic strategies and response to intervention.

**Methods:**

Clinical information was collected through structured interviews with the patient and family, teacher reports, standardized assessment tools (Conners-3, ADHD Rating Scale), and medical records. A longitudinal follow-up was conducted in a Child and Adolescent Psychiatry Unit.

**Results:**

The patient presented with long-standing symptoms of inattention, distractibility, and impulsivity, leading to significant academic underachievement and frequent classroom disruptions. At home, parents reported difficulty following rules, emotional outbursts, and frequent conflicts with siblings.

Treatment involved a multimodal approach:

- Pharmacological intervention with stimulant medication, resulting in partial improvement in attention and reduction of hyperactive behaviors.
- Psychoeducation and parent training to improve consistency in behavioral management at home.
- School-based support including individualized academic adjustments and behavioral reinforcement strategies.
- After several months, noticeable improvements were observed in classroom participation, impulse control, and self-esteem, although emotional regulation difficulties persisted.

**Conclusions:**

This case highlights the impact of ADHD on multiple areas of a child’s life and the need for a comprehensive, multidisciplinary approach. Combining medication, family-based interventions, and school support was effective in improving functioning. Continued follow-up is essential to address residual symptoms and to support the child’s developmental trajectory.

**Disclosure of Interest:**

None Declared

